# ASSOCIATION OF SYSTEMIC IMMUNE-INFLAMMATION INDEX ON THE RISK OF STROKE IN AMERICAN ADULTS

**DOI:** 10.1093/geroni/igae098.3294

**Published:** 2024-12-31

**Authors:** Xichenhui Qiu, Yiqing Huang, Changning Wei, Qun Wang

**Affiliations:** Shenzhen University, Shenzhen, Guangdong, China (People’s Republic); Shenzhen University, Shenzhen, Guangdong, China (People’s Republic); Shenzhen Polytechnic University, Foshan, Guangdong, China (People’s Republic); Shenzhen University, Shenzhen, Guangdong, China (People’s Republic)

## Abstract

**Background:**

Chronic inflammation is associated with various health consequences, including cardiovascular diseases and cancer. The systemic immune-inflammation index (SII) and system inflammation response index (SIRI) have emerged as novel inflammatory and prognostic markers. However, the relationship between SII, SIRI, and stroke risk in representative populations remains understudied. This study aimed to investigate the association between SII, SIRI, and stroke risk in US adults.

**Methods:**

A cross-sectional investigation utilized datasets from the National Health and Nutrition Examination Survey (NHANES) conducted between 2005 and 2018. Multivariate regression models were employed to assess the relationship between SII, SIRI, and stroke risk. Nonlinear relationships were described using smoothing curves and a two-tailed linear regression model. Subgroup analysis and interaction tests were performed to assess relationship stability.

**Results:**

The study included 36,129 adults aged 20 to 85 years. The analysis revealed a significant positive association between stroke risk and both SII (OR = 1.27, 95% CI = 0.98 to 1.65, p for trend = 0.0438) and SIRI (OR = 1.49, 95% CI = 1.13 to 1.97, p for trend= 0.0016) after adjusting for confounding factors.

**Conclusion:**

Elevated levels of SII and SIRI are associated with an increased risk of stroke in US adults. These findings underscore the importance of managing inflammatory indices as potential interventions to mitigate stroke risk.

